# Clients’ Emotional Experiences Tied to Therapist-Led (but Not Client-Led) Physiological Synchrony during Imagery Rescripting

**DOI:** 10.3390/e23121556

**Published:** 2021-11-23

**Authors:** Jessica Prinz, Eshkol Rafaeli, Jana Wasserheß, Wolfgang Lutz

**Affiliations:** 1 Department of Clinical Psychology and Psychotherapy, University of Trier, 54296 Trier, Germany; s1jawass@uni-trier.de (J.W.); lutzw@uni-trier.de (W.L.); 2 Department of Psychology, Bar-Ilan University, Ramat Gan 5290002, Israel; eshkol.rafaeli@gmail.com

**Keywords:** imagery rescripting, physiological synchrony, electrodermal activity, actor–partner interdependence models

## Abstract

Imagery rescripting (IR), an effective intervention technique, may achieve its benefits through various change mechanisms. Previous work has indicated that client–therapist physiological synchrony during IR may serve as one such mechanism. The present work explores the possibility that therapist-led vs. client-led synchrony may be differentially tied to clients’ emotional experiences in therapy. The analyses were conducted with data taken from an open trial of a brief protocol for treating test anxiety (86 IR sessions from 50 client–therapist dyads). Physiological synchrony in electrodermal activity was indexed using two cross-correlation functions per session: once for client leading and again for therapist leading (in both cases, with lags up to 10 s). The clients’ and therapists’ in-session emotions were assessed with the Profile of Mood States. Actor–partner interdependence models showed that certain client (but not therapist) in-session emotions, namely higher contentment and lower anxiety and depression, were tied to therapist-led (but not client-led) physiological synchrony. The results suggest that therapist-led synchrony (i.e., clients’ arousal tracking therapists’ earlier arousal) is tied to more positive and less negative emotional experiences for clients.

## 1. Introduction

Over the past two decades, psychotherapy researchers have demonstrated that imagery-based techniques are a very effective means of intervention for various disorders [1]. Because emotions are more strongly associated with images than with verbal thoughts, imagery-based techniques appear to activate emotions more strongly than simple conversation [1,2]. Much of the work on imagery-based techniques has centered on imagery with rescripting (i.e., imagery rescripting (IR)), an approach which was originally developed for work with clients who had undergone traumatic experiences (e.g., [3,4]). In IR, imagery is used to activate distressing memories replete with vivid sensory and emotional and cognitive content, an activation which also helps clarify unmet needs that still plague the client (e.g., [5]). The reactivated experience is then “rescripted” (i.e., changed in the imagination in a positive, desired direction) so that the unmet needs of the vulnerable or traumatized self are satisfied, at least in part. To accomplish this, the client is asked to imagine the scene from the perspective of their present self and step into the image to do whatever is necessary to satisfy the needs of their vulnerable selves [6,7,8].

Mental images simulate perceptual processes and elicit reactions that are quite similar to real experiences [9,10]. Consequently, as numerous laboratory studies have shown, imagery can activate strong physiological responses (e.g., [11,12,13]). To date, however, the role of physiological arousal vis-à-vis emotional activation in IR has received little attention.

This appears to be an important lacuna. After all, physiological data can serve as an objective measure of the arousal component of participants’ emotional responses, particularly those of anxiety and stress [14,15]. They can be collected with minimal client burden and disruption to the treatment itself [14]. Unlike self-reports, physiological measures can be recorded continuously with a high temporal resolution and are therefore able to capture important nuanced responses. Consequently, physiological measures may open a window into identifying additional mechanisms of change in IR that go beyond cognitive accounts (e.g., [6,16,17]) and may allow us to detect beneficial emotional processes.

The evocative potency of IR often leads to emotional activation (and to its attendant physiological reactions) in the therapist alongside the client. It is quite possible that this synchronous activation could serve as a mechanism of change by increasing the sense of a shared experience within the dyad and by catalyzing the client’s intrapersonal emotional processes.

To date, interpersonal processes in IR have received mostly theoretical attention. Rafaeli et al. [18] hypothesized that therapists’ emotional activation during IR may serve as a mechanism of change having to do with shared emotions, shared focus and greater empathy or attunement. More generally, Koole and Tschacher’s In-Sync model [19] postulated that synchronous (i.e., shared) emotions and experiences from clients and therapists lead to a shared experience and better client emotion regulation.

Several studies have investigated the dynamics and clinical meaning of client–therapist physiological synchrony (for a review, see [20]). As a group, these studies generally demonstrate positive associations between synchrony on the one hand and adaptive processes (e.g., empathy or attachment) on the other. To our knowledge, only two studies have examined physiological synchrony specifically during imagery interventions (with or without rescripting). Both studies involved multiple sessions and used electrodermal activity (EDA) as an index of arousal (based on extensive work demonstrating the sensitivity of EDA to emotional and cognitive processing, such as in [21,22]). Additionally, both studies compared client–therapist EDA synchrony during imagery vs. cognitive behavioral (CB) segments of sessions and found the former (but not the latter) to be tied to therapeutic bond ratings [23] and to the next session’s (as well as overall treatment’s) outcome [24].

Notably, both studies utilized an overall synchrony index (namely cross-correlation functions computed using ±10-s lags on the dyads’ residualized EDA time series). As such, they do not allow us to distinguish between synchronous experiences that are led by one party (e.g., the client) or by the other (e.g., the therapist). In other words, these studies’ results could not be used to identify which party typically drives the physiological synchrony, nor could they tell us whether both therapist-driven and client-driven synchrony—or only one of the two—were tied to adaptive therapeutic processes.

To begin answering these questions, we may draw on the study of synchrony processes within other dyadic contexts. In developmental psychology, for example, studies on caregiver–infant interactions have demonstrated that both parties co-regulate their physiology and emotions in a dynamically responsive way to each other [25]. Feldman (e.g., [26,27]; see also [28]) described caregiver–infant synchrony as reciprocal processes (on the sensory, hormonal and physiological levels) and suggested that it serves as an important part in infant’s development, such as in self-regulation capacity.

Several authors (e.g., [29]) have argued that synchrony between therapists and clients is likely to lead to similar self-regulation benefits. This may be particularly true for synchrony experienced during emotionally charged therapeutic moments, such as IR segments.

Specifically, in IR, the client and the therapist deliberately activate a memory or an experience which elicits an emotional reaction. Sharing and processing this experience, as well as communicating the emotions that accompany it, often generates emotional reactions in both parties, setting off dynamic dyadic affective processes [29]. Such processes may be characterized by synchrony or asynchrony, as well as by leading or following. Synchrony may exert its benefits best when a therapist tracks their client’s arousal levels—which may suggest empathic accuracy or a shared experience (e.g., [30])—or when the client tracks their therapist’s arousal level, which may suggest that co-regulation is occurring (e.g., [31]).

To examine these two (not necessarily incompatible) possibilities, the present study used EDA data from 50 client–therapist dyads who participated in a 6-session imagery-based treatment addressing test anxiety. Sessions 3 and 4 of the protocol use the traditional IR of past situations. We used the EDA data from these two sessions to investigate the associations between a client’s emotional experience during these sessions and both therapist-led and client-led physiological synchrony. Specifically, we calculated two synchrony indices. Therapist-led synchrony was defined as the cross-correlation within a lag of 0–10 s with the therapist preceding the client, while client-led synchrony was defined as the cross-correlation within a lag of 0–10 s with the client preceding the therapist. We expected our data to provide a conceptual replication of the positive associations between physiological synchrony and adaptive therapy processes found previously [23,24]. Moreover, our central (though exploratory) goal was to distinguish between therapist-led and client-led physiological synchrony and determine whether either or both are associated with adaptive emotional experiences (i.e., lower negative emotions and higher positive ones) in IR sessions.

## 2. Materials and Methods

### 2.1. Clients

A total of 90 potential participants were recruited using flyers and a campus newsletter. The following inclusion criteria were applied: (1) a score higher than 54 on the Test Anxiety Inventory (TAI [32]); (2) absence of an imminent risk for suicide; and (3) currently no other psychological treatment for test anxiety. Based on these criteria, three participants were excluded. Twelve additional participants dropped out after the intake examination because of timing or setting concerns. Seventy-five clients met the inclusion criteria and began treatment. Of these, 11 clients dropped out during the treatment period. Thus, 64 clients completed the entire 6-session protocol. The present study utilized data solely from Sessions 3 and 4, in which traditional IR techniques were used. Physiological data from 14 clients (or their therapists) were lost due to poor signals or technical problems; thus, the final sample consisted of 50 clients (44 female, MAge = 25.3, SDAge = 6.17). The clients differed in terms of their academic fields, with psychology, law, education, business and computer science being the most frequent ones. Table 1 provides additional client information.

Written informed consent was obtained from all clients. Other than receiving the treatment at no cost, participants were not compensated in any way. All procedures performed in this study involving human participants were in accordance with the ethical standards of the institutional or national research committee and with the 1964 Declaration of Helsinki and its later amendments or comparable ethical standards. This study was approved by the local research ethics committee (Nr. 01/2020, ethics committee of Trier University).

### 2.2. Therapists, Treatment, Training and Supervision

Twenty-two therapists treated between one and six clients each (M = 2.27 clients, SD = 1.09). The therapists were either psychotherapy trainees with at least one year of experience as clinicians (N = 5) or masters students in clinical psychology with no prior clinical experience (N = 17). All therapists received intensive training in using the treatment protocol and were supervised by a senior therapist in weekly video-based group supervision.

The treatment used was based on a six-session protocol integrating different cognitive behavioral (CB) as well as imagery-based techniques (for the full protocol, see www.osf.io/hraqd (accessed on 21 November 2021)). This protocol’s effectiveness for the treatment of test anxiety has been previously reported [33]. The protocol’s CB components include psychoeducation at the beginning of treatment, discussion of present and elaboration of alternative perceptions, feelings, behaviors and cognitions in past and future learning and exam situations and the optimization of learning strategies and procedures in exams. The protocol’s imagery-based components differ from session to session. The present study uses data from Sessions 3 and 4, in which the traditional IR of past situations was used.

### 2.3. Measures

#### 2.3.1. POMS

To assess the clients’ and therapists’ in-session emotions, we used a shortened version of the Profile of Mood States [34]. Each of the seven mood variables (contentment, vigor, calmness, anxiety, depression, anger and fatigue) was assessed by three items (e.g., for depression: hopeless, discouraged and sad). Clients and therapists were asked to rate the extent to which they had felt these feelings during the session on a five-point Likert scale (“1” = “not at all” and “5” = “extremely”). The POMS has been validated and applied in several previous studies (e.g., [34,35]), and it demonstrated excellent internal consistency in present study (α ranged from 0.80 (Session 3) to 0.81 (Session 4)).

#### 2.3.2. Client–Therapist EDA Synchrony

EDA was monitored simultaneously for the client and therapist. Two Ag/AgCl electrodes were attached to the third and fourth digits of the non-dominant hand. The signal was recorded at a sampling rate of 500 Hz using a Becker Meditec EDA module amplifier (Karlsruhe, Germany; with 0–100 µS, sensitivity: 25 mV/µS) connected to the acquisition computer via a Cesys C028149 USB-ISOLATOR and downsampled to 25 Hz. Several preprocessing steps were conducted before the cross-correlation functions (CCFs) were applied. First, the raw data were screened, and any artifacts were removed. Nonresponsive signals (EDA > 1 µs in at least 10% of the time series) were excluded from the analyses. Second, the signal was recorded in 1-s intervals and averaged across 2-min segments. Third, the auto.arima function (forecast package for R [36]) was applied to remove the autocorrelated component for each EDA time series. (For a similar approach, see [23,24])

Two CCFs were computed per session, one in which the client led (with a maximum lag of +10 s) and one in which the therapist led (with a maximum lag of −10 s). The maximal positive (in-phase) correlations (one for each CCF) were used as the two synchrony indices.

A total of 86 sessions (out of 50 × 2 = 100) were analyzed. Three sessions were not recorded due to technical problems. An additional 11 sessions were excluded because of non-responsive signals from either the clients or the therapists.

### 2.4. Data Analysis

#### Actor–Partner Interdependence Models

**Figure 1:**
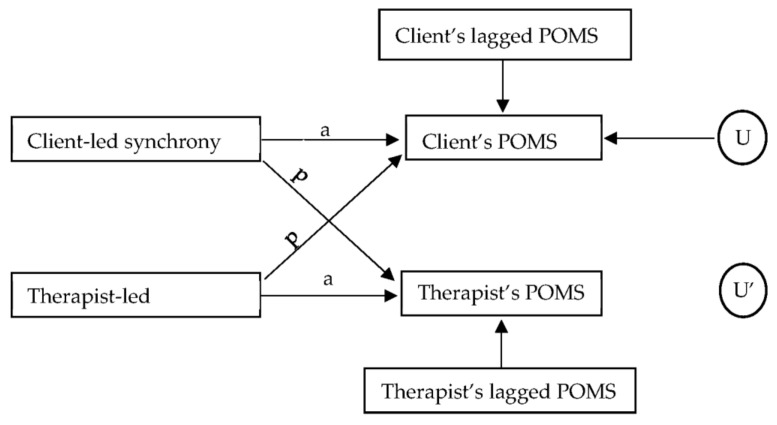
The Actor–Partner Interdependence Model. Note: U = residual error for client; U’ = residual error for therapist; a = actor effect; p = partner effect.

Given the dyadic nature of our data, we used a series of actor–partner interdependence models (APIMs; see Figure 1), with one for each POMS scale. The two dependent variables (client’s POMS ratings and therapist’s POMS ratings) are modeled on four independent variables (client-led and therapist-led physiological synchrony, as well as lagged client and therapist POMS ratings from the previous session). Paths marked as “a” represent actor effects (i.e., the degree to which the client-led or therapist-led synchrony predicts their own post-session POMS ratings). Paths marked as “p” represent partner effects (i.e., the degree to which the client-led or therapist-led synchrony predicts their partner’s post-session POMS ratings). The actor and partner paths are estimated simultaneously while adjusting for each actor’s lagged POMS ratings, and U and U’ denote the residual error terms for the two dependent variables.

The models were estimated using the two-intercept approach to multilevel modeling [37] with the following equation:
POMS_tp/cd_ = b0_cd_ + b1_cd_ × POMS_(t−1)cd_+ b2_cd_ × Client-led Synchrony_tcd_ + b3_cd_ × Therapist-led Synchrony_tcd_ + e_tcd_+ b4_pd_ + b5_pd_ × POMS_(t−1)pd_+ b6_pd_ × Therapist-led Synchrony_tpd_ + b7_pd_ × Client-led Synchrony_tpd_ + e_tpd_


Here, the POMS score in each session (t) for the client (c) or psychotherapist (p) in each dyad (d) is modeled using their own lagged (t − 1) POMS score, as well as the two (client-led and therapist-led) synchrony scores (which serve as actor and partner effects, interchangeably) and a within-person residual error score. This model was run seven times: once for each POMS emotion (contentment, vigor, calmness, anxiety, depression, anger and fatigue).

## 3. Results

The results from the three APIM analyses predicting positive emotions showed that only therapist-led synchrony was associated with clients’ emotional experience, significantly so for contentment and marginally for vigor and calmness. Client-led synchrony was not significantly associated with therapists’ or clients’ emotional experiences. All results are presented in Table 2.

### Negative Emotions as Outcomes

The results from the four APIM analyses predicting negative emotions showed that higher therapist-led synchrony was significantly associated with a lower client emotional experience of anxiety and depression, as well as marginally lower fatigue. No associations were found for the emotional experience of anger. In addition, client-led synchrony was significantly associated with the higher client emotional experience of anxiety. All results are presented in Table 3.

## 4. Discussion

The present study aimed to identify specific EDA dynamics in client–therapist dyads during IR that are associated with in-session emotional experiences. To our knowledge, this is the first study examining physiological synchrony in regard to a leading or following partner in IR. Therapist-led synchrony was significantly associated with clients’ in-session emotional experiences of greater contentment, lower anxiety and lower depression and marginally associated with the experience of greater vigor and calmness and lower fatigue. In addition, client-led synchrony was significantly associated with their own greater feelings of anxiety. No association was found between either synchrony score and the emotional experience of anger.

As expected, the results highlight the importance of distinguishing between therapist-led and client-led synchrony. Therapist-led synchrony was associated with more positive and less negative in-session client emotional experiences. In contrast, with one exception (i.e., greater anxiety), client-led synchrony was unrelated to the clients’ emotional experiences.

These results are in line with the previous literature conferring a mood-regulatory role on the therapist (e.g., [29]). Specifically, the results of the present study indicate that beneficial emotion regulation occurs not only because clients share their emotions with their therapists (as has been predicted, for example, by the social baseline theory [38]) but also because they synchronize their arousal levels with those of their therapists (but only when the temporal sequence has the therapist in the lead and is client-following). This is particularly interesting because this process typically happens outside of awareness. During the emotionally intensive IR segments, therapists often empathize with their clients’ narratives [23] and, presumably, the more this occurs, the better they are able to help the client process and regulate their emotions. The present results suggest that more effective therapists may actually be “guiding” their clients through the emotions that arise during IR, with the therapists being slightly ahead of their clients’ emotional responses.

In the present study, only certain emotions were significantly associated with therapist-led synchrony. This finding may have to do with the specific distress with which clients in this study were contending, namely test anxiety. Test anxiety is characterized by negative thoughts about consequences or failure in exams or other evaluation-related situations. Its symptoms include both emotional ones (e.g., fear and anxiety) and behavioral ones (e.g., sleep disturbance, procrastination, impaired motivation and rumination). Notably, these behavioral symptoms are quite similar to depressive reactions. This may explain why therapist-led physiological synchrony was associated most strongly with feelings of anxiety and depression.

Methodologically, the marginal findings (with fatigue, vigor and calm) and the non-significant finding (with anger) may be attributable to floor effects, namely ratings that were low and lacked much variability. Such ratings make the detection of significant effects nearly impossible.

### Strengths, Limitations and Future Directions

The current study is novel in several respects. To our knowledge, it is the first study to examine leading and following with regard to physiological synchrony in psychotherapy, rather than simply examining the overall cross-correlations. Its use of multiple (in this case, two) sessions per dyad is an additional strength, as it its focus on IR segments (in which emotional activation tends to be most consequential). By focusing on IR, a technique without eye contact, our results demonstrate that the observation of others’ emotional responses is not needed for synchronization or its benefits.

These strengths notwithstanding, several limitations of this study are noteworthy. The POMS items were used to assess emotions experienced during the entire session and not only during the IR segments. Even though the CB segments, which tended to be more conversational and less experiential, are likely generate lower emotional activation than the IR segments (see also [1,2]), these segments may have also influenced the post-session POMS ratings.

Additionally, prior research on IR mechanisms has mostly relied on laboratory studies, which induce memories or feelings in a controlled setting (e.g., [39,40]). To better understand how IR can address the emotional beliefs and dysfunctional schemas that underlie emotional disorders, we chose to examine IR mechanisms in a more ecologically valid treatment setting. However, our choice robs us of the possibility (available in lab studies) to control both the content and the temporal aspects of the experience.

Another shortcoming of the present study is its focus on sympathetic arousal. Our use of EDA, an indicator of sympathetic activation, is considered to be positively associated with emotional arousal and specifically with emotions such as anger, anxiety and fear [41]. The use of alternative measures which were unavailable to us (e.g., heart rate variability (HRV), a measure of parasympathetic functioning) may have painted a different picture, as such measures are more strongly associated with self-regulation [42]. Indeed, our non-significant results (with most positive emotions) may stem from this limitation, as our EDA responses did not capture parasympathetic functioning. Furthermore, the indexation of synchrony as a maximum positive (in-phase) correlation only reflected co-regulation to a limited extent. Future studies could benefit from the collection of both the EDA and HRV channels as well as the separate investigation of in-phase and anti-phase correlations.

## 5. Conclusions

The present study adds to the growing body of research investigating IR mechanisms, and the findings highlight the importance of examining physiological processes. The results provide the first evidence that therapists’ physiological reactions—and their clients’ subsequent synchronization with these reactions—may be tied to better session-level outcomes, at least with respect to client anxiety and depression. Methodologically, this study provides further evidence for the importance of assessing leading vs. following in physiological synchrony.

## Figures and Tables

**Table 1:** Client characteristics.

	M	SD
Academic Year	4.82	3.82
TAI	65.24	7.78
Marital Status	N	%
Single	35	70
In a Relationship	12	24
Married	2	4
Divorced	1	2
Degree Being Pursued		
Bachelor	36	72
Master	6	12
Other	8	16

**Table 2:** APIMs of therapist- and client-led synchrony as predictors of both actor and partner positive POMS ratings.

POMS Emotion	Synchrony Predictors	Estimate	Std. Error	*p*
Contentment				
	Therapist-Led Actor	6.048	3.891	0.122
	Client-Led Actor	−3.428	3.859	0.376
	Therapist-Led Partner	10.083	3.893	0.010
	Client-Led Partner	1.993	3.859	0.606
Vigor				
	Therapist-Led Actor	3.169	3.122	0.313
	Client-Led Actor	1.420	3.087	0.646
	*Therapist-Led Partner*	*5.557*	*3.113*	*0.076*
	Client-Led Partner	3.928	3.085	0.205
Calmness				
	Therapist-Led Actor	4.747	3.093	0.143
	Client-Led Actor	−2.400	3.057	0.441
	*Therapist-Led Partner*	*5.840*	*3.087*	*0.075*
	Client-Led Partner	−0.009	3.057	0.997

Note: Actor effects involve therapist-led or client-led synchrony predicting the actor’s own emotional experience as the outcome. Partner effects involve therapist-led or client-led synchrony predicting the partner’s emotional experience as the outcome.

**Table 3:** APIMs of therapist- and client-led synchrony as predictors of both actor and partner negative POMS ratings.

POMS Emotion	Synchrony Predictors	Estimate	Std. Error	*p*
Anxiety				
	Therapist-Led Actor	−2.924	3.172	0.358
	Client-Led Actor	9.383	3.120	0.003
	Therapist-Led Partner	−8.439	3.147	0.008
	Client-Led Partner	−0.299	3.142	0.924
Depression				
	Therapist-Led Actor	−1.872	3.618	0.606
	Client-Led Actor	3.790	3.580	0.292
	Therapist-Led Partner	−10.499	3.616	0.004
	Client-Led Partner	1.976	3.564	0.580
Anger				
	Therapist-Led Actor	−0.792	3.109	0.799
	Client-Led Actor	2.298	3.058	0.454
	Therapist-Led Partner	−0.999	3.084	0.746
	Client-Led Partner	0.151	3.079	0.961
Fatigue				
	Therapist-Led Actor	−3.586	3.276	0.275
	Client-Led Actor	0.192	3.244	0.953
	*Therapist-Led Partner*	*−5.908*	*3.272*	*0.073*
	Client-Led Partner	−3.012	3.268	0.358

Note: Actor effects involve therapist-led or client-led synchrony predicting the actor’s own emotional experience as the outcome. Partner effects involve therapist-led or client-led synchrony predicting the partner’s emotional experience as the outcome.

## Data Availability

The datasets analyzed in the current study are available from the corresponding author on reasonable request.
